# Design of X-Band Circulator and Isolator for High-Peak-Power Applications

**DOI:** 10.3390/mi15070916

**Published:** 2024-07-16

**Authors:** Tao Tang, Xiexun Zhang, Maged A. Aldhaeebi, Thamer S. Almoneef

**Affiliations:** 1 College of Electronic and Information, Southwest Minzu University, Chengdu 610225, China; tangt@swun.edu.cn (T.T.); zhangxiexun@foxmail.com (X.Z.); 2 Electrical Engineering Department, College of Engineering, Prince Sattam Bin Abdulaziz University, Al-Kharj 11942, Saudi Arabia; m.aldhaeebi@psau.edu.sa

**Keywords:** X-band circulator–isolator, high peak power, cascade connected, thermal analysis

## Abstract

This paper presents a design of a X-band circulator–isolator for handling high-peak-power applications. The device consists of two cascade-connected ferrite circulators, with one dedicated to transmission and the other to small-signal reception coupled with high-power signal isolation. To improve the power capacity, a layer of poly-tetra fluoroethylene (PTFE) film is placed above and below the circulator’s and the isolator’s center conductors. Measurement results show that the device is capable of withstanding a peak power of 7000 W, with an insertion loss of <0.3 dB at the transmitting port. Similarly, it sustains a peak power of 6000 W with an insertion loss of <0.5 dB at the reception port. Moreover, the proposed design achieved isolation between the transmitting and receiving ends of >20 dB with a VSWR < 1.2 at each port. Thermal analysis shows that the maximum relative ambient temperature rise is 15.11 ${\;\;}^{\circ }$C. These findings show that the proposed device achieves low-loss transmission of high-peak-power signals in the transmit channel and reverse isolation of high-peak-power signals in the receive channel.

## 1. Introduction

An example of a nonreciprocal device is a circulator, which consists of a ferrite substrate and a central component. The substrate is made of gyromagnetic ferrite, which is affected by a combination of a DC-biased magnetic field and a one-way transmission characteristic [1,2,3].

When a three-port circulator is connected to a load that matches, it functions as an isolator. The isolator utilizes the Faraday rotation effect, where an electromagnetic wave passing through a gyromagnetic ferrite with an external DC bias magnetic field causes the polarization plane to rotate. This results in the polarization plane being perpendicular to the grounding resistance plate during forward transmission, leading to minimal attenuation, and parallel to the grounding resistance plate during reverse transmission, resulting in almost complete absorption of the electromagnetic wave [4]. For many applications, the circulator and isolator design must adhere to the following design specifications: temperature, size, insertion loss, isolation, bandwidth, and VSWR [5].

High-power signals are frequently utilized in applications such as electronic countermeasures, radar detection, and electromagnetic energy transmission. With peak powers reaching the kilowatt range, these signals challenge the power endurance capabilities of transmission and reception equipment. In the design of circulators for such high-power applications, it is imperative to prioritize factors including isolation, insertion loss, bandwidth, temperature stability, and matching loads, as these are critical for assessing circulator performance [6,7].

The Y-junction configuration, commonly utilized in circulator and isolator construction, features a central biased ferrite material, integral to the three-component Y-junction circulator design. The circulators’ nonreciprocal function arises from the ferrite material’s nonlinear response to high inputs, leading to elevated heating and increased power losses. However, the need for operation within precise input power ranges restricts their application in high-power scenarios, due to increased power losses, heating, and non-linear behavior [8,9,10].

This study introduces a design for a circulator and isolator device, which consists of two interconnected circulators that facilitate signal transmission and reception for high-power applications, ensuring superior reverse isolation. The design integrates the second circulator’s input port, which serves as a highly isolated receiving port due to its coupling with a matched load that effectively absorbs reverse power, with the first circulator’s output port acting as a high-power transmission unit. By strategically dispersing heat sources and employing an independent compartmentalized structure, the design effectively addresses the thermal challenges associated with high-power operation. Additionally, the device maintains excellent electromagnetic compatibility, as detailed in the third section of this paper.

## 2. Designing Principle

**Figure 1:**
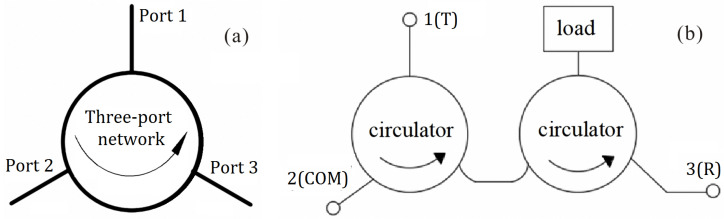
Schematic diagram of the three-port circulator and the cascaded-connection circulator and isolator: (**a**) three-port circulator, (**b**) cascaded-connection circulator and isolator.

Figure 1a shows a diagram of a three-port, nonreciprocal, passive circulator. The circulator operates on the following principle: When a signal is input through port 1, with both port 2 and port 3 being matched, the signal will be output through port 2 while port 3 will have no signal output. Similarly, if the signal is input through port 2, with both ports 1 and 3 being matched, the signal will be output through port 3 and no signal will be output through port 1 [11].

Connecting port 1 to a matching load creates an isolator, with the level of isolation depending on the matching between port 3 and the RF matching load, where the degree of isolation is contingent upon the quality of the match between port 1 and the matching load.

Only the low-field working mode, which operates with a low magnetic field strength beneath the resonant magnetic field, can be utilized by the X-band circulator [12]. The initial radius of the center ferrite disc is determined by the following formula [13]:

(1) $$R=\frac{K\lambda_{0}}{2\pi \sqrt{\varepsilon_{e}\mu_{e}}},$$

where *K* is a constant with a 1.5–1.8 range. $\varepsilon_{e}$ and $\mu_{e}$ represent ferrite’s effective permittivity and relative permeability, respectively. The wavelength of the circulator’s operating center frequency in free space is denoted by $\lambda_{0}$. And the effective permeability of the ferrite is given by [14]

(2) $$\mu_{e}=1-\rho^{2},$$

The normalized saturation magnetization of ferrite is represented by ρ=γ0·4πMsγ0·4πMsf0f0, where $\gamma_{0}$ is the gyromagnetic ratio and the saturation magnetization $4\pi M_{s}$ and the circulator’s operating center frequency $f_{0}$ define its value. Given the design’s peak power needs, a ferrite substrate with a saturation magnetization of 1800 Gs and a relative dielectric constant of $\varepsilon_{e}$ = 14.2 and a spin-wave line width of $\Delta H_{k}>$ 30 Oe was selected.

Utilizing Equation (1), the initial calculation yields a central ferrite disc diameter of approximately 6.8 mm at a frequency of 8 GHz. Furthermore, since the ferrite heat dissipation is poor and the peak power through the circulator can reach several kilowatts, it is advisable to increase the thickness of the ferrite substrate to enhance the ferrite’s resistance to the breakdown electric field. This will raise the circulator’s peak power capacity. Additionally, to enhance the ferrite substrate’s heat dissipation area and the ferrite layer’s heat conduction efficiency, the diameter of the ferrite substrate should also be increased as much as feasible.

## 3. Results and Discussion

Figure 1b depicts the connection mode of the suggested design, which cascades two three-port circulators. Circulator 1, positioned on the left in Figure 1b, operates as a signal transmitter, following the circulator’s operating principle. The transmitting signal enters through port 1 (labeled 1(T)) of circulator 1. Due to the limitations of the circular path, the signal is exclusively transmitted from port 2 (labeled 2(COM)) to the connected antenna.

For high-power signal reception and reverse isolation, one of circulator 2’s ports (the right circulator in Figure 1b) is connected to a matched load capable of high-power absorption. Circulator 1 and circulator 2 together comprise the receiving channel since the signal that the antenna receives enters through port 2 of circulator 1 and exits through port 3 (labeled 3(R)) of circulator 2.

A limiter and the subsequent circuit are connected to port 3 of circulator 2. The limiter is triggered and reflects the high-power signal if the received signal power exceeds a safe threshold. The reflected signal is then absorbed by the matched load through the isolator.

**Figure 2:**
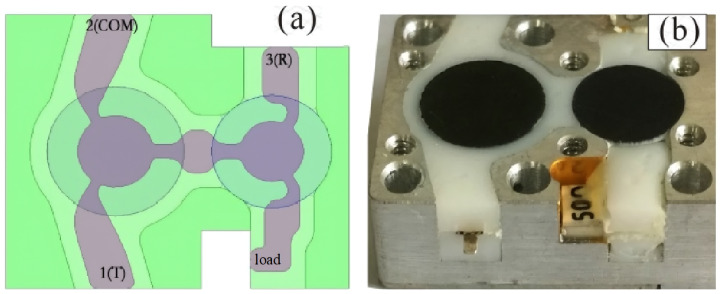
Structure drawing of cascaded-connection circulator and isolator: (**a**) top view of internal structure, (**b**) photos of the prototype.

Figure 2a,b display the simulation model and prototype of the cascaded circulator and isolator, respectively, with dimensions of 20×17×7 mm${\;\;}^{3}$. Following optimization of the electromagnetic and thermal properties, the circular ferrite substrate’s diameter in the center of the circulator measures 8 mm, while the circular ferrite substrate’s diameter in the center of the isolator measures 7 mm.

It is worth mentioning that all the simulations were conducted using the commercial full-wave simulator Computer Simulation Technology (CST) [15].

**Figure 3:**
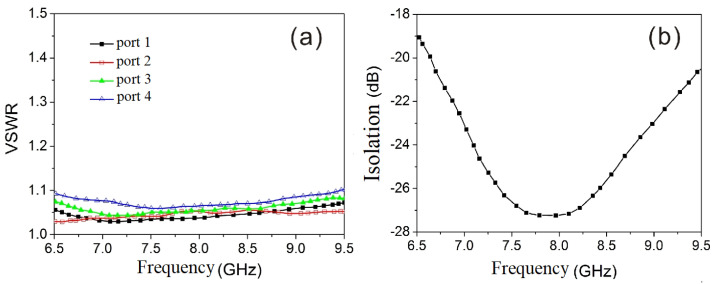
VSWR of each port and isolation between receiving and transmitting ports: (**a**) VSWR, (**b**) isolation.

The circulator uses a single Y disc junction circuit in its classical version [16], which has a higher power resistance, a bigger operating bandwidth, and the lowest insertion loss. Furthermore, as illustrated in Figure 3a, the signal transmission lines are filleted at every corner. In regions with high electric field intensity, abrupt angles and small filleted corners are avoided, which can enhance the product’s peak power resistance. Figure 2a depicts the matching relationship between the ports in the simulation model and those in Figure 1b.

**Figure 4:**
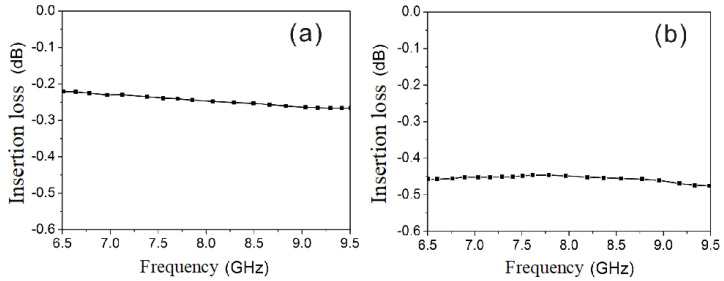
Insertion loss of transmission and receiving channels: (**a**) transmission channel, (**b**) receiving channel.

Figure 3a presents the VSWR simulation results for each port, and Figure 3b illustrates the isolation between the transmitting and receiving ports (ports 1 and 3). It is evident that each circulator and isolator port has a VSWR of <1.2 and that there is >20 dB of isolation between the transmitting and receiving ports. Figure 4a,b display the insertion loss for both the transmitting and receiving channels. It is evident that the receiving channel has an insertion loss of ≤0.5 dB, whereas the transmitting channel has an insertion loss of ≤0.3 dB.

As seen in Figure 2a, a smooth design is chosen for the connection between the ports and the central disc drive to lessen signal reflection at the connection sections. The area with a concentrated electric field is filled with condensed silica gel, which offers exceptional resistance to temperature extremes, aging, electrical insulation, moisture, and shock, as well as superior adhesion. This measure is taken to prevent potential breakdown in the circulator and isolator.

The use of condensed silica gel, with a high relative dielectric constant (typically ≥ 2.8), also enhances the peak power capacity of these components. To further effectively stop the breakdown between the central conductor and the ferrite substrate, a layer of PTFE film with a thickness of 0.05 mm is affixed to the top and bottom surfaces of the central conductor in both the circulator and isolator.

**Figure 5:**
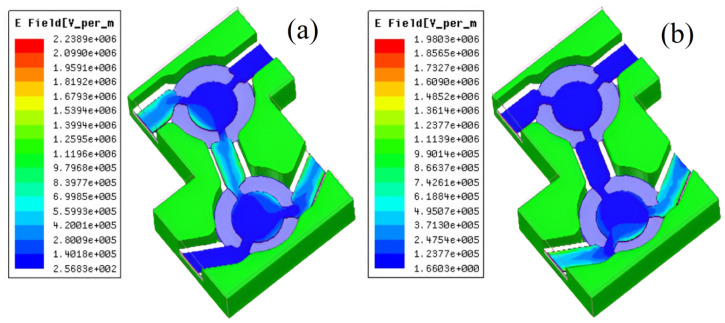
Electric field distribution of central conductor of the proposed design: (**a**) transmission channel, (**b**) receiving channel.

Due to the high power requirements of the circular and isolator, the design’s electric field intensity distribution was thoroughly examined. Figure 5 shows the values for the field intensity distribution on the transmitting and receiving channels’ center conductor surfaces. As shown in Figure 5, the maximum electric field intensity on the surface of the transmitting channel’s central conductor is $E_{max}\leq 2.24\times 10^{6}$ V/m when the peak power input from the transmitting port 1(T) is 7000 W, and the maximum electric field intensity on the receiving channel’s surface is $E_{max}\leq 1.98\times 10^{6}$ V/m when the peak power input from the antenna end 2(COM) is 6000 W.

Prior to attaching the PTFE film layer, the maximum electric field strength is $2.75\times 10^{6}$ V/m and $2.50\times 10^{6}$ V/m, respectively. Both the transmitting and receiving channels have a maximum electric field intensity that is less than the threshold value $E_{b}\leq 3\times 10^{6}$ V/m, which represents the breakdown electric field intensity of air.

## 4. Thermal Analysis and Measurement Results

Temperature affects electronic component reliability in two ways: (1) when the component junction temperature goes above permissible limits, the component fails to function; (2) component expansion or contraction during heating results in thermal stress and deformation of the metallized ceramic substrate and heat sink. It is necessary to regulate the temperature rise of equipment since it directly affects both the dependability and the failure rate of electronic components. The most popular application of the Arrhenius equation is to represent how temperature affects the failure rate, as provided by [17,18]:

(3) $$F=A\exp\left(-E_{a}/kT\right)$$

where *F* is the rate of failure, *T* is the temperature (K), *k* is Boltzmann’s constant, *A* is a constant, and $E_{a}$ is the activation energy (eV). Equation (3) illustrates how the failure rate of electronic components rises exponentially with temperature; for instance, if activation energy $E_{a}$ = 0.65 ${\;\;}^{\circ }$C is chosen, the failure rate will double for every 10 ${\;\;}^{\circ }$C increase in temperature.

**Figure 6:**
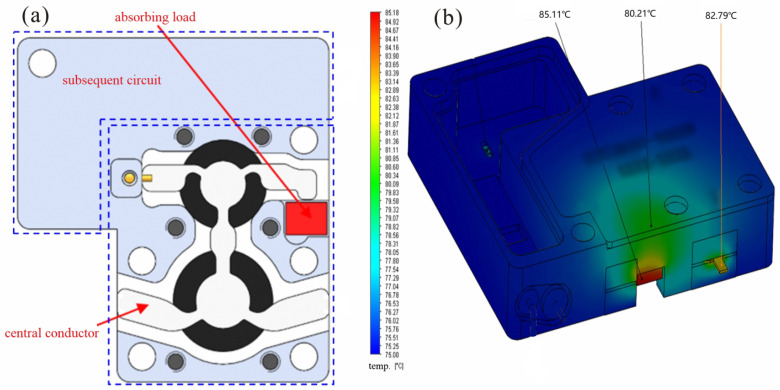
Showing both heat source distribution and temperature distribution: (**a**) main heat source, (**b**) temperature distribution.

The system’s thermal analysis and design must be completed to guarantee that it can operate steadily and dependably and lower its failure rate. Figure 6a illustrates the distribution of heat sources within the circulator–isolator, while Figure 6b displays the temperature distribution of the components once the system has reached an equilibrium state. The metal shell temperature is kept constant at 75 ${\;\;}^{\circ }$C in this investigation, whereas the ambient temperature is considered to be 70 ${\;\;}^{\circ }$C. Heat conduction, heat convection, and heat radiation are the parameters used in the simulation.

The central conductor and matched load in the suggested circulator and isolator serve as the primary heat sources. The temperature on the corresponding load is 85.11 ${\;\;}^{\circ }$C, the temperature on the central conductor is 82.79 ${\;\;}^{\circ }$C, and the temperature on the surface shell above the load is 80.21 ${\;\;}^{\circ }$C, as seen in Figure 6b, once the temperature reaches equilibrium. Put another way, while the system is operating normally, the highest temperature it may reach is the temperature across the matched load, and both the relative shell temperature and the relative ambient temperature climb to 10.21 ${\;\;}^{\circ }$C and 15.11 ${\;\;}^{\circ }$C, respectively.

The aluminum alloy’s high thermal conductivity (155 W/m.K.), light weight, strength, and reliable sealing make it ideal for the component package shell. Inside, a gold-plated W-Cu alloy carrier, which includes the matched load, is soldered. The W-Cu alloy’s low thermal expansion and high thermal conductivity (150–220 W/m.K.) further enhance heat management. Effective thermal conduction from the heat source, aided by the dispersed heat source distribution and moderate individual heat consumption, addresses the heat dissipation challenge under normal conditions.

In microwave equipment, electromagnetic compatibility design is especially crucial. The intended component uses an independent cavity’s layout, and circuits like circulators and isolators, as well as the limiter that follows, use independent cavities’ plug-and-in and plug-out designs to create a whole.

Lastly, Table 1 displays the measurement findings of the constructed prototype of the suggested circulator–isolator operating in the 8–9 GHz range.

An input power of 0 dBm was utilized to measure the VSWR, then insertion loss and the isolation. And the results show that the maximum VSWR of each port is <1.2; the insertion loss from 1(T) to 2(COM), is ≤0.3 dB, and that from 2(COM) to port 3(R) is ≤0.5 dB; and the isolation between the transmitting and receiving ports is >20 dB.

This high-power device was designed to operate steadily at 7000 W in transmit mode and 6000 W in receive mode, as confirmed by simulations. The constructed prototype was measured with input powers of 7000 W and 6000 W (the input single with a pulse width of 10 microseconds and a duty cycle of 0.4% with a peak power 7000 W and 6000 W) at ports 1(T) and 2(COM), respectively, mirroring real-world use. The experimental results show the device functioning as expected under such high input powers.

Table 2 shows a performance comparison between the proposed device and some previous devices in the literature, such as VSWR, return loss, isolation, and size. Despite not outperforming all competitors in Table 2, our proposed X-band circulator–isolator stands out for its specialized design that addresses the needs of high-power environments. It provides good isolation and maintains low insertion loss. The use of PTFE film within our device enhances power capacity. Through a thoughtful layout and careful material selection, our device can effectively manage the heat dissipation challenges that are commonly encountered in high-power scenarios. The dual functionality of our design, serving as both a circulator and an isolator, provides a unique advantage over the existing literature, making it a comprehensive solution for high-power applications.

## 5. Conclusions

This paper introduces an innovative design for high-power signal transmission and reception, employing two cascaded circulators. The design excels in high-power handling and isolation, with distinct roles for each circulator: one for transmission and the other for signal reception and isolation. The system is capable of withstanding peak powers of 7000 W in transmission and 6000 W in reception, with minimal insertion losses and excellent VSWR and isolation performance. Thermal analysis indicates that the maximum system temperature aligns with the matched load’s temperature, which is associated with the isolator.

## Figures and Tables

**Table 1:** Five manufactured prototype samples of the suggested wideband circulator and isolator were used to gather experimental data.

No.	Parameters		Unite	Measurement Conditions	Desired Value	Measured Results
1	VSWR	1(T)	/	8–9 GHz, input power 0 dBm	≤1.2	1.18
		2(COM)	/		≤1.2	1.16
		3(R)	/		≤1.2	1.15
2	insertion loss	1(T) to 2(COM)	dB		≤0.3	0.28
		2(COM) to 3(R)			≤0.5	0.49
3	isolation	2(COM) to 1(T)	dB		≤−20	−22.5
		3(R) to 2(COM)			≤−20	−20.1
4	power	1(T)	W	7000	≥7000	work steady
		2(COM)		6000	≥6000	work steady

**Table 2:** Comparison with previous related works.

Reference	Work Model	VSWR or Return Loss	Isolation (dB)	Insertion Loss (dB)	Size
Ref. [8]	circulator	22 dB (return loss)	−22	0.2	/
Ref. [9]	circulator	18 dB (return loss)	−17	0.3–0.6	0.86λ×0.72λ
Ref. [14]	circulator	/	−20	0.2	/
Ref. [17]	circulator	20 dB (return loss)	−20	0.1	/
**This work**	**Circulator and isolator**	**≤1.2 (VSWR)**	**≤−20**	**≤0.3 & ≤0.5**	**20×17×7 mm${\;\;}^{3}$**

## Data Availability

The original contributions presented in this study are included in the article; further inquiries can be directed to the corresponding author.
